# Development and *Ex Vivo* Assessment of Topical Formulations for Delivery of Stigmasterol and β‑Sitosterol

**DOI:** 10.1021/acsomega.6c00333

**Published:** 2026-07-10

**Authors:** Dominique Mesquita e Silva, Carolina Neves Cunha, Maria Esther Leal da Silva Sad, Giovanna Ferreira Paiva dos Santos, Guilherme Diniz Tavares, Fernanda Maria Pinto Vilela, Juliana de Carvalho da Costa

**Affiliations:** † Department of Pharmaceutical Sciences, 28113 Federal University of Juiz de Fora, Juiz de Fora, MG 36036-900, Brazil

## Abstract

Phytosterols are sterol compounds naturally produced by plants and fungi, known for their ability to interact with steroid receptors and promote anti-inflammatory effects. This study aimed to investigate their potential for topical application by developing and assessing formulations containing stigmasterol (STIG) and β-sitosterol (β-SIT). A gel-cream and a topical film were prepared, and an HPLC-UV-DAD method was developed and validated for the simultaneous quantification of both phytosterols. *Ex vivo* skin penetration was evaluated using porcine ear skin mounted on vertical Franz diffusion cells. The analytical method demonstrated high selectivity, linearity, precision, and accuracy, with satisfactory recovery in spiked skin samples, and the penetration study showed that β-SIT incorporated into the topical film achieved significantly higher deposition in the viable epidermis and dermis compared with the gel-cream (44.16 ± 19.69 vs 17.65 ± 15.68 μg/cm^2^; a 2.5-fold increase). These findings highlight the potential of phytosterol-based topical systems for the development of innovative therapies targeting skin inflammation.

## Graphical abstract

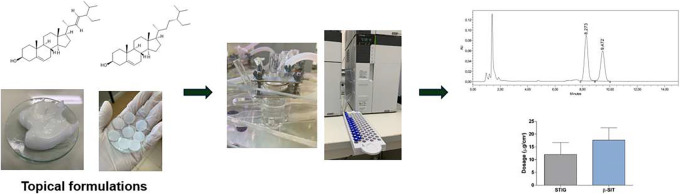

## 1 Introduction

Phytosterols are a group of sterols produced exclusively by plants and fungi, playing key roles in biological processes such as membrane structure and fluidity, hormonal biosynthesis, and responses to biotic and abiotic stress.^1,2^ They are commonly found in seeds and grains, such as soybeans, olives, cotton seeds, and lotus seeds,^3^ as well as in medicinal plants like *Pereskia aculeata*,^4^ *Bambusa bambos* ^5^ and *Rhizophora mucronata*.^6^

Stigmasterol (STIG) and β-sitosterol (β-SIT) are the primary phytosterols present in plant cell membranes. These molecules share a similar chemical structure, differing only by the presence of an unsaturation in the side chain of STIG^7,8^ (Figure 1). Their structural similarity to endogenous steroid hormones allows them to interact with steroid receptors, functioning as agonists or antagonists and thereby conferring a range of biological activities.^9^ The most well-documented properties of STIG and β-SIT include antihyperglycemic, antihypercholesterolemic, antioxidant, antiviral, and anti-inflammatory effects.^10,11^

**Figure 1:**
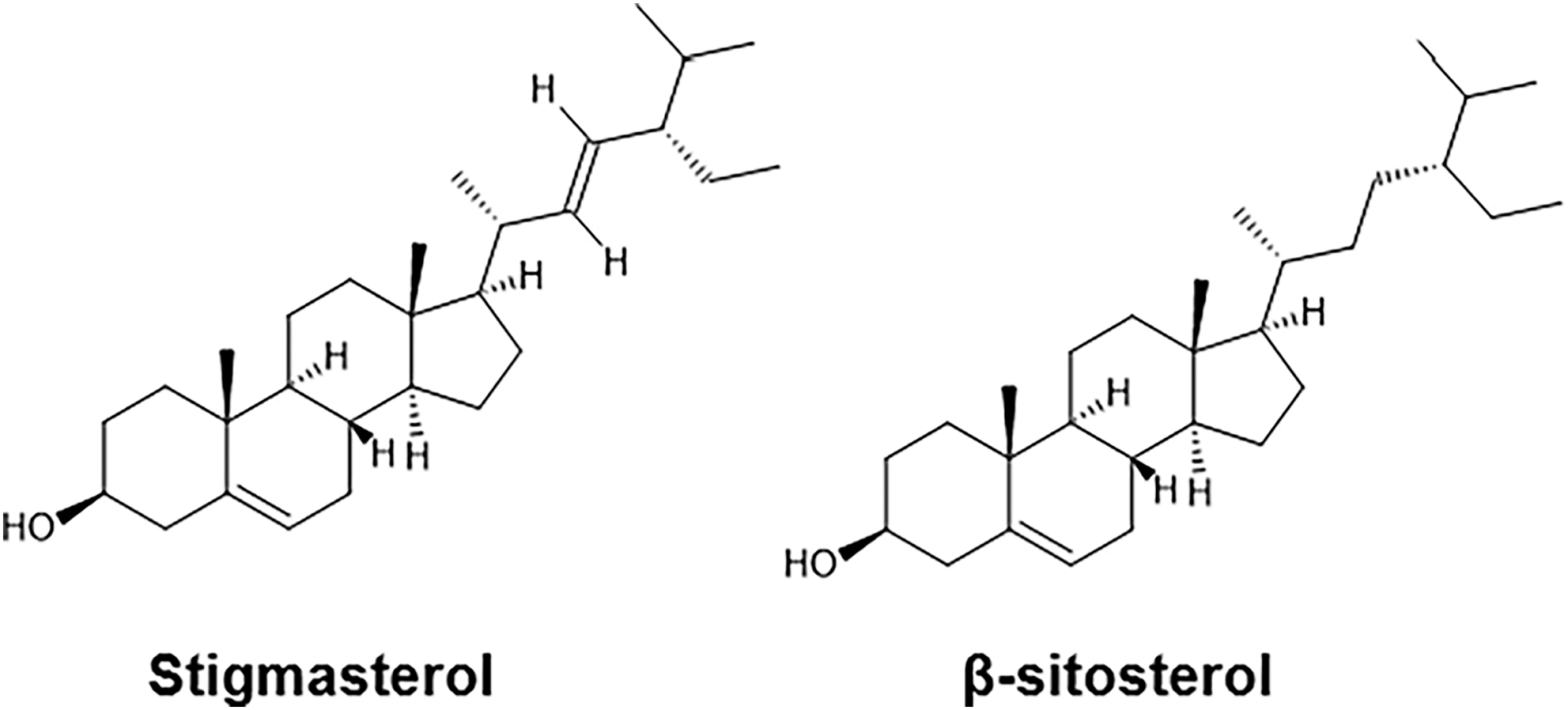
Chemical structure of phytosterols, STIG and β-SIT.

Several studies have demonstrated the anti-inflammatory activity of phytosterols. According to Akinloye (2020),^12^ these metabolites can selectively inhibit cyclooxygenase-2 (COX-2) via hydrogen bonding and downregulation of COX-2 mRNA expression. Additionally, they reduce the expression of inflammatory mediators such as interleukin-6 (IL-6) and inducible nitric oxide synthase (iNOS).^13^

In the context of developing phytosterol-based topical formulations with anti-inflammatory potential, the technological development phase is crucial.^14^ This phase involves characterizing physicochemical properties, assessing compatibility with excipients, studying solubility and bioavailability, standardizing the formulation, and performing quality control, safety, and efficacy evaluations.^15^

For dermatological applications, skin permeation and penetration studies using vertical Franz diffusion cells are widely employed, as they allow investigation of how pharmaceutical forms influence the distribution of active compounds through skin layers.^16^ Thus, understanding skin transport mechanisms is essential for optimizing topical drug delivery.^17^

Given the therapeutic potential of phytosterols in modulating inflammation, this study aimed to develop topical formulations containing STIG and β-SIT, and to evaluate their skin penetration using *ex vivo* diffusion models.

## 2 Materials and Methods

### 2.1 Chemicals and Reagents

The phytosterols, STIG and β-SIT, were purchased from Sigma-Aldrich (Brazil). The following excipients and reagents were used in the formulation development: glycerin, menthol, Optiphen, silicone DC200/350, butylhydroxytoluene (BHT), polyethylene glycol 400 (PEG 400), polysorbate 80 (Tween 80), and Dry-Flo, all obtained from Fagron (São Paulo, Brazil). Hostacerin SAF was supplied by PharmaSpecial (Campinas, Brazil); propanediol was purchased from Engenharia das Essências (Brazil); pullulan was acquired from Attivos Magistrais (São Paulo, Brazil); and Germall 115 was obtained from Chemyunion (São Paulo, Brazil). HPLC grade methanol was supplied by J.T.Baker (Barueri, Brazil), and deionized water was produced using a Milli-Q Plus purification system (Millipore, USA).

### 2.2 Determination of the Physicochemical Properties of Phytosterols

The prediction of physicochemical properties was performed using SwissADME, following the methodology described by Bakchi.^18^ The evaluated parameters included molecular weight (MW), partition coefficient (log *P*), number of hydrogen bond donors and acceptors (H-bonds), and skin permeability coefficient (log *K_p_*).

### 2.3 Gel-Creams Preparation

Four gel-cream formulations (GC1–GC4) were prepared without the application of heat, as summarized in Table 1. The concentrations of excipients varied among the formulations to enable selection of the most appropriate composition; however, the preparation method remained consistent across all formulations. Initially, menthol, butylated hydroxytoluene (BHT), and Dry-Flo were triturated. This was followed by levigation with propanediol. Subsequently, the remaining components Optiphen, silicone DC200/350, and Hostacerin SAF were incorporated. Purified water was then added under continuous stirring to achieve a gel-cream consistency. Finally, the pH was measured and adjusted to 4.5–5.0 using a 20% citric acid solution. Following the preparation of the gel-cream base, 6% STIG and 6% β-SIT (w/w) were incorporated.

**Table 1:** Composition of the Tested Gel-Cream Formulations^b^

components % (w·w^–1^)	F1	F2	F3	F4
Hostacerin SAF	8	8	4	6
Glycerin	2	5		
Propanediol			2	5
Dry-Flo	3			
Silicone DC 200/350		2	1	2
Optiphen	1	1	1	1
Menthol	0.5	0.3		0.2
BHT			0.05	0.05
Purified water, sq^a^	100	100	100	100

a sq: Sufficient quantity for the final volume.

b Lists the concentrations of excipients used in the four gel-cream formulations (GC1–GC4) prepared at room temperature to optimize spreadability, stability, and incorporation of phytosterols.

### 2.4 Topical Films Preparation

Six types of topical film formulations (TF1–TF6) were developed by varying the concentrations and types of polymers. The films were prepared using a pharmaceutical laminator (FagronLab), following the solvent casting method described by Ferreira.^19^ The combinations of polymers tested are detailed in Table 2. The preparation procedure for the selected formulation involved the following steps: Pullulan was first dispersed in purified water, followed by the sequential addition of PEG 400, propanediol, imidazolidinyl urea, and menthol. The pH was measured after complete solubilization of the components. Lamination was then performed on the surface of a glass plate. The films were dried in an oven at 40 °C for 30 min (Fanem, 315 SE-Primo). After drying, the films were removed from the oven and cut into 6 cm × 5 cm sections. Following the selection of the optimal topical film formulation, 6% β-SIT was incorporated into the pharmaceutical film using the same preparation procedure described previously.

**Table 2:** Composition of Tested Topical Film Formulations^a^

components % (w·w^–1^)	F1	F2	F3	F4	F5	F6
Pullulan				10	12	14
PVA	5	7	7			
HPMC	2		2			
PEG 400		4	2	4	2	2
Glycerin	5			5		
Propanediol	2	2	2	2	2	2
T80	2	2	2	2	2	2
Germall 115	0.3	0.3	0.3	0.3	0.3	0.3
Menthol	0.2	0.2	0.2	0.2	0.2	0.2
Purified water, qsp	31.50	34.50	34.50	26.50	31.50	29.50

a Details the polymer types, plasticizers, and auxiliary ingredients used to prepare six topical film formulations (F1–F6) by the solvent casting technique.

### 2.5 Analytical Method Development and Validation

Phytosterols were quantified using a high-performance liquid chromatography (HPLC) system equipped with a photodiode array detector (Waters E2695 with PDA 2998 UV–vis detector). The method was adapted from Delgado-Zamarreño^20^ and validated according to the parameters recommended by the Brazilian Health Regulatory Agency,^21^ including selectivity, linearity, intra- and interassay precision, limit of detection (LOD), limit of quantification (LOQ), and recovery of spiked samples.

Chromatographic separation was performed on an Eclipse Plus C18 reverse-phase column (4.6 mm × 100 mm, 5 μm; Agilent) using an isocratic mobile phase composed of methanol and water (98:2, v/v). The flow rate was set at 0.9 mL/min, with an injection volume of 50 μL. The column oven was maintained at 25 °C, detection was carried out at 210 nm, and the total run time was 15 min.

#### 2.5.1 Selectivity

Selectivity was assessed by comparing the chromatographic profiles of STIG and β-SIT standards with those of the mobile phase, formulation components, and skin matrix. No interfering peaks were observed at the retention times of STIG and β-SIT, confirming the selectivity of the method. Peak resolution (Res) was calculated according to eq 1.

1 Res=2(tr⁡2−tr⁡1)/(Wb1+Wb2)

in which: tr = retention time, Wb = width of the base.

#### 2.5.2 Linear Range

The analytical calibration curve was constructed using standard solutions of STIG and β-SIT in methanol at an initial concentration of 200 μg/mL. Six serial dilutions were prepared to obtain final concentrations ranging from 3.125 to 100 μg/mL. Linear regression analysis was performed, yielding a linear equation in the form *y* = *ax* + *b*, where *a* is the slope and *b* is the intercept. Linearity was evaluated based on the linear correlation coefficient (*r* ^*2*^). All measurements were performed in triplicate.

#### 2.5.3 Limit of Detection and Limit of Quantitation

The theoretical LOD and LOQ were calculated based on the standard deviation of the response (σ) and the slope of the calibration curve (S), using data from three independent calibration curves. LOD and LOQ were calculated using eqs 2 and 3, respectively

2 LD=3.3×σ/S

3 LQ=10×σ/S

The LOD and LOQ were experimentally confirmed by analyzing chromatograms obtained from the injection of standard solutions at their respective limit concentrations. These concentrations were subsequently incorporated into the average calibration curve to ensure accurate quantification at the low concentration levels required for the skin penetration studies.

#### 2.5.4 Precision and Accuracy

Precision was evaluated in terms of repeatability (intra-assay) and intermediate precision (interassay). Intra-assay precision was assessed by analyzing six replicates of standard solutions at three concentrations (10, 30, and 90 μg/mL) on the same day under identical experimental conditions. Interassay precision was evaluated by analyzing the same concentrations on two different days. Precision was expressed as the relative standard deviation (RSD%), calculated according to eqs 4 and 5.^22^

Accuracy was assessed based on the degree of agreement between the measured values and the true (nominal) values of the analytes. It was expressed as a percentage of recovery at each concentration level, along with the corresponding RSD%.

4 $$s=\frac{\sqrt{\sum {\left(\mathrm{xi}-\mathrm{xm}\right)}^{2}}}{n-1}$$

5 $$\mathrm{RSD}\;\left(\%\right)=\frac{s}{\mathrm{xm}}\times 100$$

Which:*xm*: Average of determinations; *xi*: Individual value of a measurement; *n*: Number of measurements

#### 2.5.5 Recovery of STIG and β-SIT from Skin Matrix

The applicability of the HPLC method and the extraction procedure for evaluating the skin penetration of STIG and β-SIT were assessed by performing recovery studies using spiked porcine ear skin samples. Circular skin sections with an area of 1.77 cm^2^ were cut and spiked with standard solutions of STIG at concentrations of 56.64, 84.96, and 113.28 μg/mL, and β-SIT at concentrations of 70.90, 113.28, and 141.60 μg/mL (*n* = 4 for each level).

Following spiking, the solvent was allowed to evaporate at room temperature. The substances were then extracted by adding 2.5 mL of methanol to each skin sample, followed by vortex mixing for 1 min and sonication in an ultrasonic bath for 30 min. The extracts were centrifuged for 10 min, and the supernatant was filtered through a 0.45 μm membrane filter prior to HPLC-PDA analysis.

Control samples consisted of porcine ear skin that had not been spiked with STIG or β-SIT. Recovery was calculated as the average percentage of analyte retrieved from the spiked samples, using eq 6.^21,22^

6 $$\mathrm{recovery}\;\left(\%\right)=\frac{\mathrm{experimental}\;\mathrm{concentration}}{\mathrm{theoretical}\;\mathrm{concentration}}\times 100$$

### 2.6 Skin Preparation

The pig ear skins were donated by a local slaughterhouse. Soon after slaughter, the ears were sent to the laboratory, where the cleaning procedure was carried out with distilled water. Subsequently, the skin was removed from the cartilage using a scalpel. The hair, blood vessels, and adipose tissue were removed from the skin, leaving only the stratum corneum (SC), epidermis (EP) and dermis (D). Only intact skin samples, without visible damage were used.^23–25^ The cleaned skins were stored under refrigeration at −4 °C and could be used for a maximum of 30 days.

### 2.7 *Ex Vivo* Skin Penetration

*Ex vivo* skin penetration testing was carried out in 12 mL static Franz-type vertical diffusion cells. The donor compartment contained the gel-cream formulation or the topical film (*n* = 6) and the receptor compartment was filled with the respective ethanol: water receptor medium (50:50). It was ensured that all air beneath the porcine skin was removed. Ethanol was included in the receptor solution to improve the solubility of the phytosterols, which are highly lipophilic compounds with limited aqueous solubility. The use of an ethanol mixture (50:50, v/v) was intended to maintain sink conditions throughout the Franz diffusion cell experiments, ensuring adequate solubilization of the permeated compounds and preventing saturation of the receptor medium, which could compromise the diffusion gradient and affect permeation results. This receptor medium composition was selected in accordance with the recommendations of the Organisation for Economic Co-operation and Development Guidance Notes on Dermal Absorption (OECD), which support the use of hydroalcoholic receptor systems for lipophilic substances to ensure appropriate recovery and reliable permeation assessmen.^26^

For the gel-cream formulation, 59 mg of the formulation with STIG and β-SIT at a concentration of 6% each were added, and as a control, 59 mg of the pure gel-cream were added, without phytosterols. The amount of gel cream formulation was calculated to achieve the same number of active substances in comparison with the application of the topical film. For the topical film formulation, the films containing 6% β-SIT were cut into areas of 1.77 cm^2^, and were added to the donor compartment. The formulations were prepared and evaluated immediately prior to the skin penetration experiments to ensure their suitability for topical application.

The receptor solution was kept under constant stirring at 500 rpm. At the end of 24 h, the excess of formulation on the skin was removed and the SC was removed using the tape stripping technique, 20 times on each skin.^27^ Soon after, the skins were cut and the same extraction process as the recovery test was carried out.

### 2.8 Statistical Analysis

The parameters evaluated in the validation of the analytical method were obtained using Waters Empower software and were treated statistically using Microsoft Excel software. Quantitative data are presented in tables and figures as mean ± standard deviation, from 9 replicates for precision and 3 replicates for the other analyses. In the statistical analysis of skin penetration tests, the Student *t* test was used followed by the Mann–Whitney U test. The level of statistical significance for all analyses was accepted as *p* < 0.05.

## 3 Results and Discussion

### 3.1 Determination of the Physicochemical Properties of Phytosterols

One of the first stages of preformulation studies is the characterization of the physicochemical properties of the substances used and the verification of compatibility with excipients.^15^ Therefore, the physical-chemical properties of STIG and β-SIT were first evaluated, such as solubility, partition coefficient, and molecular weight, factors that, in general, can influence skin penetration.^17^

The physicochemical properties were determined by molecular modeling. The difference in terms of the molecular weight of phytosterols is relatively low, with β-SIT (414.71 g/mol) having a higher molecular weight than STIG (412.69 g/mol). The molecular weight of both phytosterols was less than 500 g/mol, in accordance with the literature, in which molecules smaller than 500 g/mol cross the skin barrier with less difficulty.^17^ Lipophilicity was determined by log *P*, and the two phytosterols presented extremely high values, with β-SIT presenting the highest log *P* (7.24), not being an ideal value for topical delivery reported in the literature, where values ideal log *P* values range from 1 to 4 ^28^. Both phytosterols presented one H-bond acceptor and one H-bond donor. The numbers of H-bond acceptors and donors can be inversely correlated with skin penetration, H-acceptors ≤ 3 and H-donors ≤ 2, can be considered good penetrants.^29,30^ Furthermore, the software calculated the permeability coefficient (log *K_p_*), which predicts skin penetration according to the physicochemical properties of the compounds. β-SIT showed a higher log *K_p_* when compared to STIG, indicating greater skin permeability.^31^ Data on physicochemical properties are presented in Table 3.

**Table 3:** Physicochemical Properties of Stigmasterol and β-Sitosterol Calculated Using SwissADME Molecular Modeling^a^

compounds	molecular formula	MW (g mol^–1^)	Log *P*	H-bond acceptor number	H-bond donor number	Log *K*_p_ (cm·h^–1^)
STIG	C_29_H_48_O	412.69	6.98	1	1	–2.74
β-SIT	C_29_H_50_O	414.71	7.24	1	1	–2.20

a Legend: MW, molecular weight.; log *P*, oil/water partition coefficient; log *K_p_*, the skin permeability. Summarizes the calculated molecular weight, log *P*, hydrogen bond donors and acceptors, and predicted skin permeability coefficients for each phytosterol.

### 3.2 Formulation Development

#### 3.2.1 Gel-Cream

Gel-cream dosage forms can be a good choice for the development of topical formulations, as their constituents are quickly absorbed, in addition to the ease of incorporating substances.^32^ The aim of developing the gel-cream formulation containing STIG and β-SIT is to obtain good spreadability and hydration of the SC, to promote the efficient penetration of metabolites into the SC.^33^

The excipients were selected based on their pharmaceutical functionality, compatibility with phytosterols, and suitability for topical delivery intended to retain the actives within the superficial skin layers. Experimental formulations (GC1–GC4) were designed by varying the proportions and classes of humectants, emollients, and sensory modifiers to optimize formulation homogeneity, spreadability, and sensory performance upon application. Hostacerin SAF was selected as the self-emulsifying base due to its ability to provide adequate consistency and facilitate the incorporation of lipophilic compounds into the semisolid system.^34,35^ According to the manufacturer’s technical information, the base exhibits a broad pH stability range (4.0–9.0) and is capable of incorporating a wide variety of components.^36,37^

Glycerin and propanediol were investigated as humectants and cosolvents to enhance hydration and formulation uniformity, whereas dimethicone (Silicone DC 200/350) was incorporated as an emollient and sensory modifier to improve spreadability and reduce tackiness.^38,39^ Propanediol is a natural derivative extracted from corn, but it can also be produced synthetically, and widely used in the pharmaceutical industry as a possible substitute for propylene glycol.^40,41^ Dry-Flo (Aluminum Starch Octenylsuccinate) was evaluated to impart a dry-touch sensory profile and enhance cosmetic acceptability.^42,43^ BHT was included as an antioxidant to minimize oxidative degradation, and menthol was incorporated as a skin penetration enhancer to facilitate permeation across the stratum corneum, the primary barrier to topical delivery.^44,45^

Different concentrations of Hostacerin SAF were tested, as well as wetting agents, sensory modifiers, preservatives, and antioxidants. Immediately after preparing the formulations, the organoleptic characteristics and pH of each formulation were evaluated in Table 4. The selection criterion for the skin penetration test was based on organoleptic characteristics and visual aspects.

**Table 4:** Evaluation of the Organoleptic Characteristics and pH of the Gel-Cream^a^

formula	odor	appearance	color	visual aspects	pH
F1	Characteristic minty	Homogeneous	White	Opaque and viscous	7.2
F2	Characteristic minty	Homogeneous	White	Shiny and viscous	7.5
F3	Characteristic minty	Homogeneous	White	Shiny and fluid	6.3
F4	Characteristic minty	Homogeneous	White	Shiny and viscous	6.7

a Presents the sensory attributes (odor, appearance, color, and texture) and pH values used as criteria for selecting the most appropriate gel-cream formulation.

Formulation F4 was chosen for the incorporation of STIG and β-SIT, as it presents the best organoleptic and visual characteristics. The initial pH of the formulation was 6.7, which was adjusted to 5.0 with a 20% citric acid solution to bring it closer to the pH of the skin. To incorporate 6% STIG and 6% β-SIT into the gel-cream, it was necessary to levigate with 2% propanediol.

#### 3.2.2 Topical Film

The excipients were selected according to their film-forming capacity, mechanical performance, flexibility, and ability to modulate drug release and skin permeation. Experimental formulations (TF1–TF6) were developed by varying the composition of the polymeric matrix and the plasticizing system. Therefore, three types of polymers were selected: pullulan, hydroxypropyl methylcellulose (HPMC), and poly­(vinyl alcohol) (PVA), tested in different concentrations, together with two types of plasticizers, PEG 400 and glycerin. A total of 50 g of each polymeric solution was prepared, and the remaining components of the formulation were added. The polymeric solutions were evaluated for appearance, viscosity, and pH. Soon after, the polymeric solutions were laminated with the help of the laminator, cut into sizes of 6 cm × 5 cm, and evaluated for drying time, film formation, structural characteristics, and flexibility in Table 5.

**Table 5:** Evaluation of the Organoleptic Characteristics of Polymeric Films^a^

	polymeric solution			polymeric film (dry)			
formula	appearance	viscosity	pH	dry time (min)	film-formation	aspect	flexibility
F1	Turbid	High	6.7	50	Complete	Turbid	Nonflexible
F2	Clear	High	7.0	50	Complete	Clear	Nonflexible
F3	Turbid	High	6.9	50	Complete	Turbid	Nonflexible
F4	Clear	Low	4.9	30	Complete	Clear	Nonflexible
F5	Clear	Low	4.6	30	Complete	Clear	Nonflexible
F6	Clear	Medium	5.1	30	Complete	Clear	Flexible

a Evaluates the appearance, viscosity, drying time, film formation, and flexibility of each polymeric solution to determine the most suitable polymer–plasticizer combination.

All polymers tested were capable of forming films, but factors such as drying time, appearance of the polymeric solution, and flexibility were taken into consideration when choosing the formulation. Although glycerin has a plasticizing effect reported in the literature, it was not able to provide flexibility when used alone. The plasticizing effect was only observed with the use of PEG 400. PVA and HPMC were not the polymers of choice due to the drying time, high viscosity of the base, and the relatively laborious preparation procedure. PVA is solubilized at higher temperatures, between 70–80 °C, and takes around 12 h to solubilize, making it ideal to solubilize overnight.

The film-forming polymer of choice for the skin penetration tests was pullulan, due to the fact that it has the best characteristics, such as a translucent polymeric base, and fast drying time, in addition to the fact that it solubilizes quickly in water (around 30 min), optimizing the handling process.^46^ Furthermore, pullulan is biodegradable, nontoxic, and highly biocompatible, facilitating the incorporation of other excipients in the formulation.^47^ The addition of pullulan in formulations can enhance the healing effect, as it has been determined in animal studies that the polymer can accelerate the regeneration of skin tissue by increasing collagen synthesis, making it a good choice for anti-inflammatory formulations.^48^

Regarding the plasticizer, PEG 400 showed better performance. It has a hydrophilic nature, favoring the diffusion of water in the polymer matrix, which influences the malleability of the films and the removal of the glass plate.^49^ The menthol was maintained at the same concentration as the gel-cream to promote skin penetration.

To compare the skin penetration of STIG and β-SIT in topical pharmaceutical forms, both phytosterols were initially incorporated at the same concentration. However, the topical film presented a dosage limitation due to its fixed dimensions, which prevented proper film formation when both phytosterols were included simultaneously. Therefore, for comparative purposes, only β-SIT was incorporated into the topical film at a concentration of 6%.

### 3.3 Method Validation

#### 3.3.1 Selectivity

The selectivity of the method was evaluated by considering the components of the developed formulations and the skin (SC, EP, and D). Peak identifications were performed by comparing the pattern retention times. The retention times verified for STIG and β-SIT were 8.4 and 9.6 min, respectively, with a resolution of 2.63. Interference peaks were not identified in the skin layers and the developed formulations (Figure 2). The HPLC analytical method showed good selectivity and resolution for the evaluated phytosterols.

**Figure 2:**
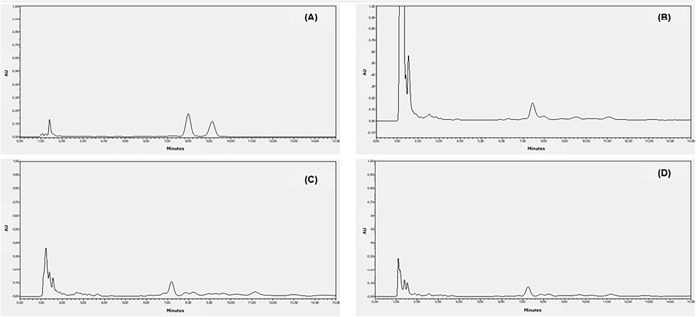
HPLC-UV chromatographic profiles of standards of stigmasterol (STIG) and β-sitosterol (β-SIT) (A), pig ear skin (B), gel-cream formulation (C), topical film formulation (D).

#### 3.3.2 Linearity, Limit of Detection, and Limit of Quantification

The analytical curves were constructed for STIG and B-SIT by plotting peak area ratios against the concentration of standard solutions. The method was found to be linear in the range of 3.125 to 200 μg/mL, showing a highly significant correlation coefficient (*r*2) of 0.9998 for both phytosterols. For quantification of STIG and B-SIT, the regression equations can be seen in Table 6.

**Table 6:** Parameters Determined from the Analytical Curve^a^

analyte	equation	*r* ^2^	LOD (μg mL^–1^)	LOQ (μg mL^–1^)
STIG	*y* = 13418x + 17064	0.9998	0.208	0.632
β-SIT	*y* = 9725x + 10136	0.9998	0.879	2.662

a Provides the regression equations, correlation coefficients (*r* ^2^), and limits of detection (LOD) and quantification (LOQ) determined during analytical method validation.

The calculation of LOD and LOQ determines the sensibility of the method.^22^ The LOD and LOQ obtained were 0.208 and 0.632 μg/mL for STIG, and 0.879 and 2.662 μg/mL for β-SIT, respectively. The values represented correspond to the smallest amounts of STIG and β-SIT that can be detected and quantified with precision and accuracy and are therefore suitable for analyzing phytosterols in the samples studied.

#### 3.3.3 Precision and Accuracy

The precision of the method was determined by repeatability (intra-assay) and intermediate precision (interassay) and represented as the coefficient of variation (CV) in Tables 7 and 8. The intra-assay and interassay precisions displayed were evaluated and CV% values were 0.52 and 2.04% for STIG, and 0.78 and 2.07% for β-SIT. According to Ribani et al.,^22^ methods that quantify compounds in macro quantities require an RSD of 1 to 2%. In methods of trace or impurity analysis, RSDs of up to 20% are accepted. According to Brasil (2017),^21^ acceptance criteria must be defined and justified according to aspects such as the purpose of the method and the concentration of the analyte in the sample. Based on these considerations, a 95% confidence interval was adopted, with RSD < 5% as the acceptance criterion for precision and the 95–105% interval for accuracy.

**Table 7:** Precision and Accuracy Values of the Analytical Method for Quantifying Stigmasterol in Methanol^a^ ^,^ ^b^

theoretical concentration (μg.mL^–1^)	experimental concentration (μg.mL^–1^)	precision RSD (%)	accuracy (%)
Intermediate precision (interassay)—Day 1(*n* = 9)			
10.0	8.60	0.81	99.32–101.68
30.0	29.47	1.76	98.08–102.86
90.0	93.94	1.17	98.47–101.85
Intermediate precision (interassay)—Day 2 (*n* = 9)			
10.0	8.55	0.52	99.50–100.67
30.0	29.14	2.00	97.24–102.71
90.0	94.23	0.73	99.57–101.58
Repeatability (intra-assay) (*n* = 9)			
10.0	8.61	0.82	99.08–101.44
30.0	29.53	1.76	97.68–102.43
90.0	94.09	1.18	99.25–102.03

a RSD = relative standard deviation.

b Reports the intra- and interassay precision (RSD%) and accuracy data obtained for stigmasterol at different concentration levels.

**Table 8:** Precision and Accuracy Values of the Analytical Method for Quantifying β-Sitosterol in Methanol^a^ ^,^ ^b^

theoretical concentration (μg.mL^–1^)	experimental concentration (μg.mL^–1^)	precision RSD (%)	accuracy (%)
Intermediate precision (interassay)—Day 1 (*n* = 6)			
10.0	8.59	0.85	99.39–101.46
30.0	34.64	1.24	98.70–101.70
90.0	103.35	0.94	99.29–101.57
Intermediate precision (interassay)—Day 2 (*n* = 6)			
10.0	8.47	0.92	97.95–100.05
30.0	34.18	0.78	98.93–101.07
90.0	102.92	2.00	96.15–101.15
Repeatability (intra-assay) (*n* = 6)			
10.0	8.59	0.86	98.50–100.04
30.0	34.49	1.25	98.33–101.32
90.0	103.29	0.95	98.92–101.19

a RSD = relative standard deviation.

b Presents the intra- and interassay precision (RSD%) and accuracy values obtained for β-sitosterol at multiple concentration levels.

#### 3.3.4 Recovery

Recovery analysis is part of the accuracy of analytical validation being an important parameter for determining the ability of the method to recover the analyte in a complex matrix, such as skin.^47^ According to Brasil (2017),^21^ to determine the accuracy of active substances, the proposed method used is called chemical reference substance (SQR). The method consists of determining the proportion of substances extracted from the skin layers relative to the amount initially added.^16^ The average recovery percentages of STIG and β-SIT are respectively, 62.62% and 91.64%, which can be seen in Table 9.

**Table 9:** Data Obtained by the Recovery of Stigmasterol in Pig Ear Skin and β-Sitosterol^a^

stigmasterol			β-sitosterol		
theoretical concentration (μg/mL)	experimental concentration (μg/mL)	recovery (%)	theoretical concentration (μg/mL)	experimental concentration (μg/mL)	recovery (%)
56.64	38.81	67.66 ± 1.26	70.08	61.47	86.82 ± 2.62
84.96	48.87	57.87 ± 0.60	113.28	104.61	92.35 ± 3.04
113.28	69.73	62.33 ± 1.23	141.06	139.31	95.76 ± 3.13
Average Recovery		**62.62 ± 4.90**	Average Recovery		**91.64 ± 4.51**

a Summarizes the mean recovery percentages and experimental concentrations of stigmasterol and β-sitosterol extracted from porcine skin matrices spiked with known amounts of each compound.

### 3.4 *Ex Vivo* Skin Penetration Study

The *ex vivo* tests were carried out using pig ear skin as a barrier for the skin penetration of phytosterols, due to its histological similarity with the skin to the human forearm.^39^ Phytosterols were applied to the skin surface and quantified after 24 h. The two formulations developed were tested: gel-cream and topical film. The quantification of compounds was monitored in two sections: (1) Concentration retained in the skin after 24 h; and (2) permeate concentration in the receptor medium. The phytosterols that remained in the SC were not quantified by the tape stripping method, since the objective of the formulation was to reach the deeper skin layers, such as viable epidermis, and dermis, where the main inflammatory mediators are found.^50,51^

In Franz cells skin penetration assays, the deposition of compounds in the donor compartment is indicative of cutaneous absorption, while the accumulated concentration in the receptor medium is used as an indicator of systemic absorption.^52,53^ Phytosterols were quantified in the viable epidermis and dermis, but in the receptor medium, it was not possible to perform quantification, as the values presented were below the detection limit.

Thinking about this aspect, the result was promising, due to the fact that the systemic absorption of phytosterols has adverse effects such as increased systolic and diastolic blood pressure and changes in the hormonal balance of testosterone and estradiol.^43,54^ The amounts of STIG and β-SIT recovered from the viable epidermis and dermis when vehiculated in the gel-cream can be seen in Figure 3.

**Figure 3:**
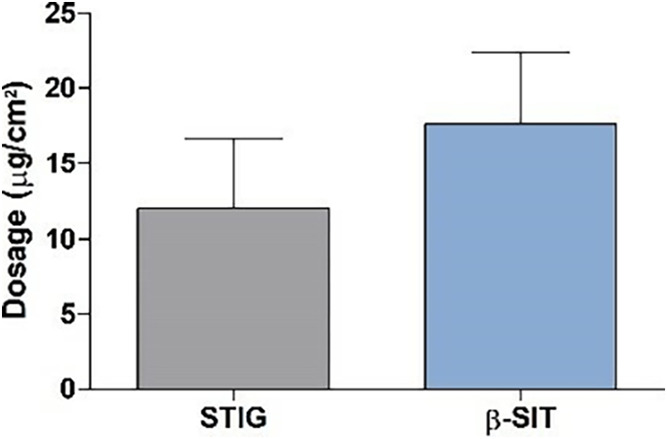
Amounts of STIG and β-SIT recovered from the viable epidermis and dermis of the gel-cream.

After 24 h of analysis, the cumulative amount of STIG and β-SIT that penetrated the viable epidermis and dermis were 12.02 ± 20.97 μg/cm^2^ and 17.65 ± 15.78 μg/cm^2^, respectively. Although the skin penetration of β-SIT was greater than that of STIG, there was no statistically significant difference between them. There are few data in the literature on skin penetration of phytosterols. A study carried out by Chang^30^ performed the skin penetration of three phytosterols in 3 mM solutions, in which the greatest skin penetrability was predicted for β-sitosterol (0.33 nmol/mg), followed by campesterol (0.21 nmol/mg) and stigmasterol (0.16 nmol/mg).

Cutaneous penetration of phytosterols tends to be challenging due to their limited physicochemical characteristics, such as high molecular size and log *P*, low solubility, high crystallinity, and hydrophobicity, making it necessary to use solvents, surfactants, and cosolvents to improve solubility and dispersion.^43,44^ The phytosterols STIG and β-SIT have log *P* 6.98 and 7.24 respectively, indicating high lipophilicity, which can cause the accumulation of these substances within the lipid phase of the SC, impairing the passage to the viable epidermis and dermis^30,55^ .

In this way, the excipients used in the formulation, as well as the pharmaceutical form of choice, can help penetrate the SC and permeate the substances into the deeper layers of the skin.^56^ To overcome the challenge of skin penetration of phytosterols given the physical-chemical characteristics presented, pharmaceutical forms such as gel-cream and topical film were compared with the skin absorption of β-SIT Figure 4.

**Figure 4:**
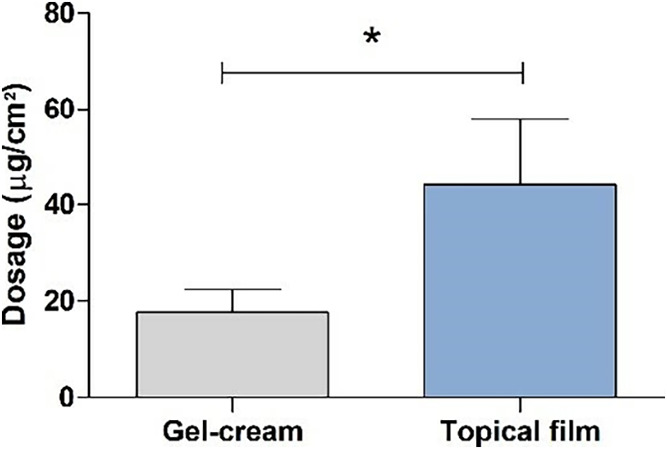
Comparison of the skin absorption of β-SIT in the pharmaceutical forms gel-cream and topical film.

The topical film demonstrated significantly greater skin penetration of β-SIT compared to the gel-cream formulation (44.16 ± 19.69 vs 17.65 ± 15.68 μg/cm^2^, respectively; *p* < 0.05). When expressed as a percentage of the applied dose over 24 h, skin penetration was 1.24% for the topical film and 0.48% for the gel-cream. In other words, β-SIT incorporated into the topical film was able to penetrate the viable epidermis and dermis approximately 2.5 times more effectively than when delivered via the gel-cream.

Based on the data presented, it was observed that the topical film achieved greater skin penetration, due to its adhesive characteristic, forming a thin polymeric layer on the surface of the skin, inducing an occlusive effect.^57^ Occlusion will reduce water loss from the skin, which will consequently favor percutaneous absorption through the expansion of intercorneocyte gaps.^58,59^ In addition to occlusion, the topical film applied to the skin is homogeneously distributed, prolonging its substantivity in the skin, which will establish a transient supersaturation of the substances, favoring skin penetration.^60,61^

Furthermore, the main component of the topical film formulation, pullulan, may have favored skin penetration. Pullulan has been widely used in targeting active substances through various topical, implant, ocular, oral, and liquid pharmaceutical forms. In topical formulations, it has the promising potential to deliver active substances to the skin.^62,63^ In the cosmetic area, recent studies have developed soluble pullulan-based microneedles to optimize the dermal delivery of molecules with healing potential.^64,65^

Another factor observed when analyzing skin penetration was the ease of application of the film when compared to the gel-cream. The film applied to the moistened skin easily adhered to the pig ear skin, forming a thin adhesive layer. The gel-cream was more difficult to apply, as a homogeneous layer was not formed. This is one of the factors that can hinder the skin penetration of semisolid pharmaceutical forms and considering a topical treatment, it may present disadvantages such as dose uncertainty, accumulation of substances in different areas, and shorter contact time with the skin.^66^

## 4 Conclusions

This study presented a promising topical formulation containing the phytosterols STIG and β-SIT, capable of penetrating the skin layers, reaching the dermis, where key inflammatory mediators are located. Notably, the topical film demonstrated enhanced skin penetration of β-SIT at the target site. However, due to dosage limitations inherent to the film’s dimensions, the simultaneous incorporation of both phytosterols was not feasible. These findings suggest the potential of this formulation as a novel therapeutic approach for the treatment of inflammatory skin conditions. Nevertheless, further investigations are required to assess the pharmacological efficacy and toxicological safety of the developed formulation in the treatment of inflammation and other dermatological disorders.
